# Supplementary material for: Improved Discriminability of Severe Lung Injury and Atelectasis in Thoracic Trauma at Low keV Virtual Monoenergetic Images from Photon-Counting Detector CT
Source: Diagnostics (Basel). 2024 Oct 6;14(19):2231. doi: 10.3390/diagnostics14192231 (PMC11475707; doi:10.3390/diagnostics14192231)

Figure S1: Discriminability of atelectasis and severe lung injury - percentage distribution of the subjective assessment score of all three raters. Responses: 1 = no differentiation, 2 = poor differentiation, 3 = mostly discriminable, 4 = good differentiation, 5 = excellent differentiation;

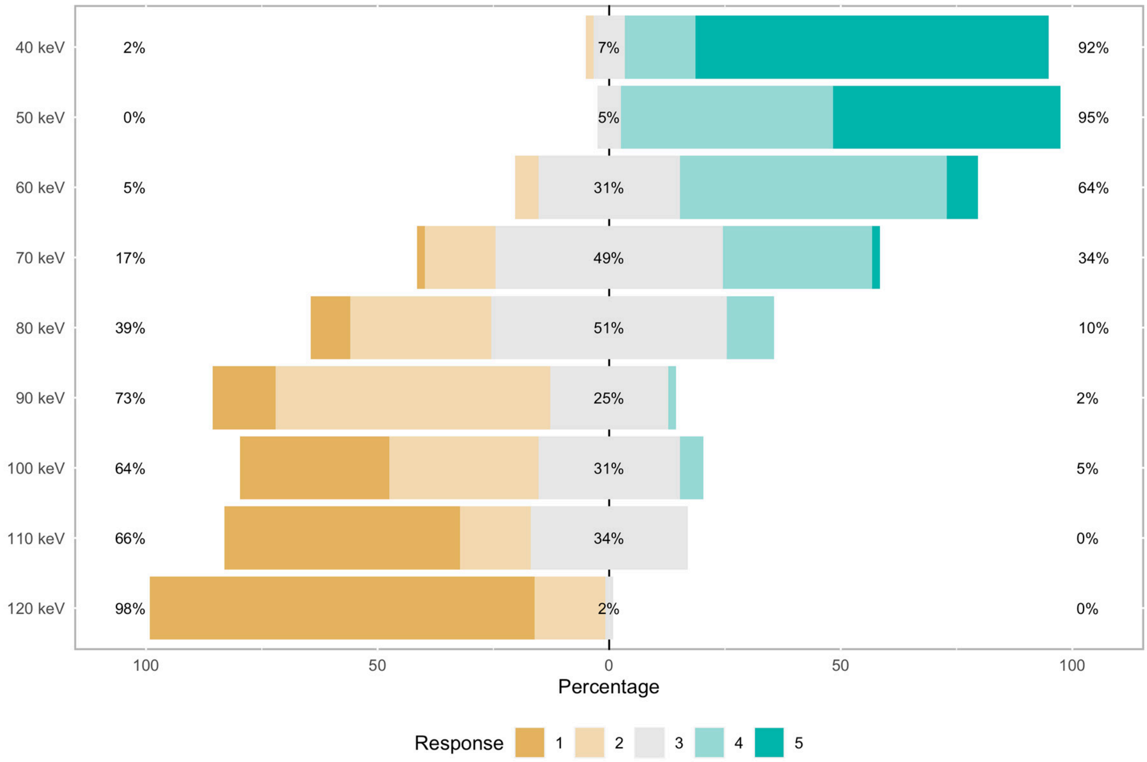

Figure S2: Image noise - percentage distribution of the subjective assessment score of all three raters. Responses: 1 = major noise, 2 = more than average noise, 3 = average noise, 4 = minor noise, 5 = insignificant noise;

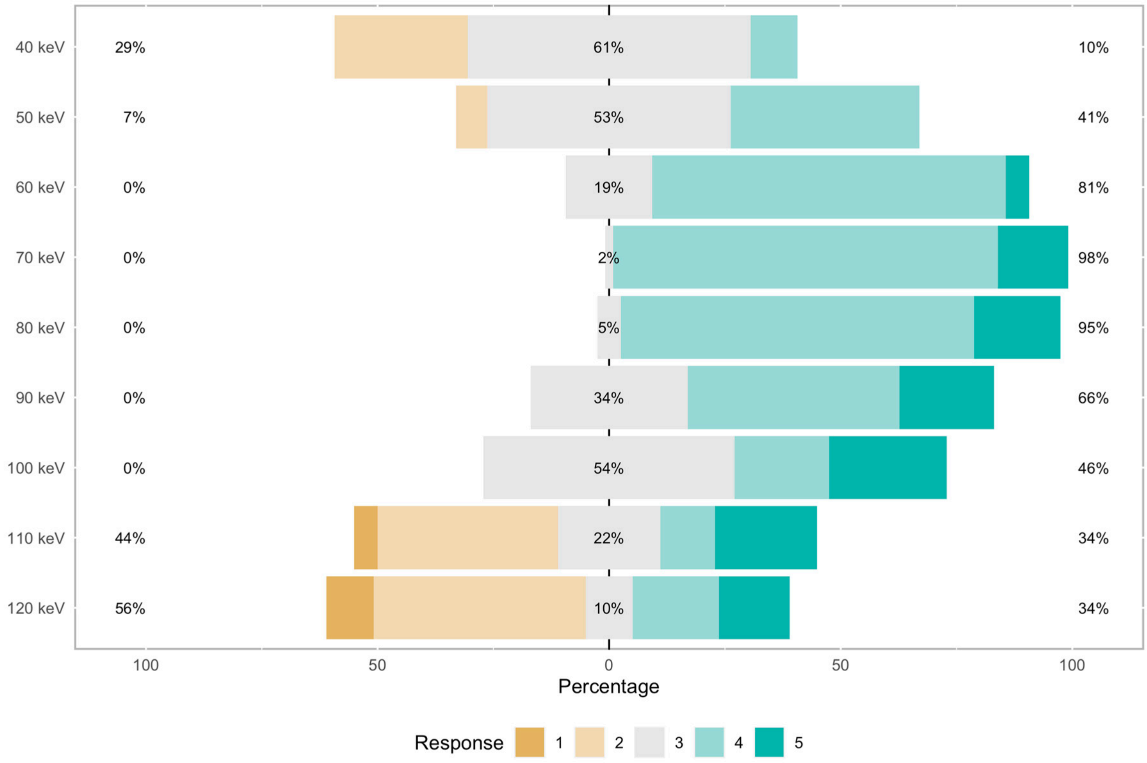

Figure S3: Overall image quality - percentage distribution of the subjective assessment score of all three raters. Responses: 1 = very poor, 2 = poor, 3 = acceptable, 4 = good, 5 = optimal.

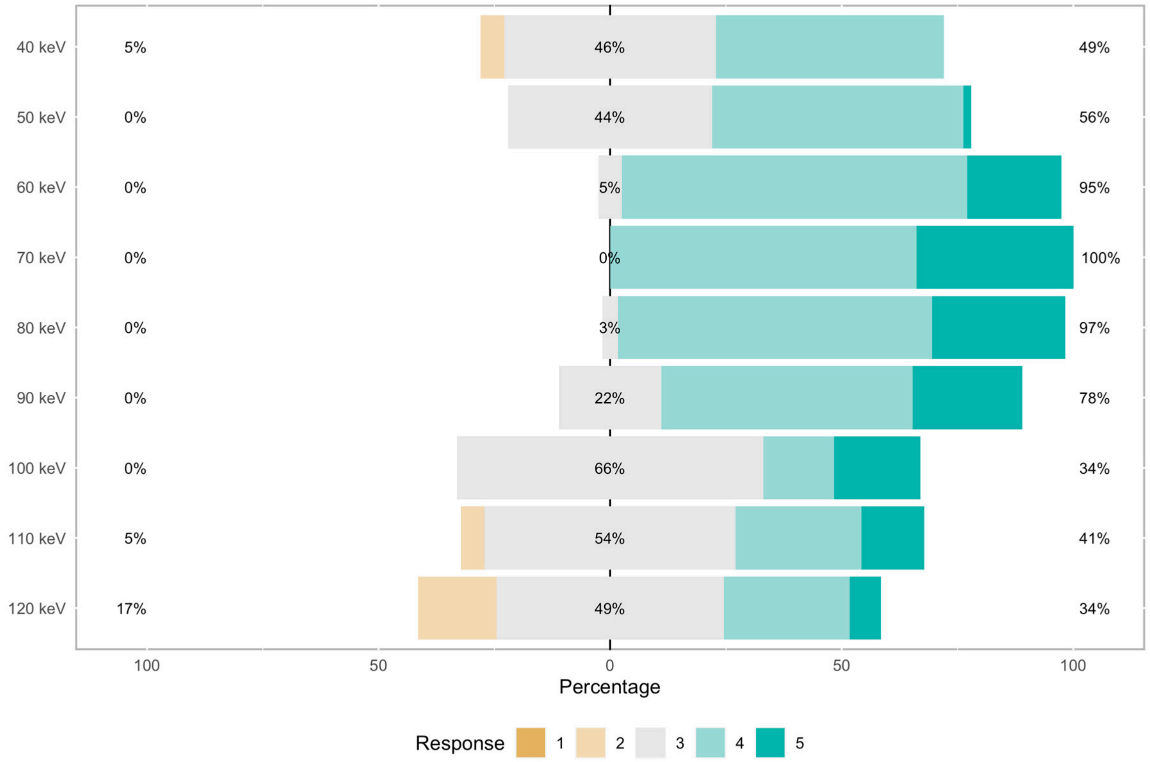

Supplement: Supplementary file 1 [file diagnostics-14-02231-s001.zip › diagnostics-3207797-supplementary.pdf]
